# An International Medical Society in Texas

**Published:** 1886-08

**Authors:** 


### ORGANIZATION OF AN INTERNATIONAL "COUNTY" MEDICAL SOCIETY IN TEXAS.

The following, clipped from a Brownsville Texas Paper is kindly sent us by our correspondent and friend Dr. A. S. Wolff.

On Saturday a meeting of the medicial men of Brownsville, Texas, and Matamoros, Mexico, was held at Dr. Wolff's office. All the medical gentlemen of both sides of the river were in attendance, which proves the alacricty with which they responded to the call of Dr. Wolff and the desire that animates them to form an organized society, such as exists in many cities and districts where there are less medical men and less necessity of these social and professional organizations. The following is the roster of our medical men:

Dr. Bentley, U. S. A., Dr. A. S. Wolff, Dr. C. B. Combe, Dr. Et. Melot, Dr. Main, Dr. Valls, Dr. I. Martinez, Dr. C. Macmanus, Dr. M. Cisero, Dr. Dias Gutierrez, Dr. M. Barragan, Dr. Manuel Carpio, Dr. Guzman and Dr. Ortega.

Dr. Macmanus was voted to the chair and Dr. Valls as secretary for the occasion. On permanent organization Dr. Macmanus was unanimously elected permanent president Dr. M. Cicero, Vice-president, Dr. Valls secretary, Dr. C. B. Combe treasurer and Drs. Melou and Cicero censors.

After having passed a pleasant hour in discussing their organization and future proceedings, it was agreed to have the next meeting of the society in Matamoros on the 15th.

